# Nutrient-Dense Shelf-Stable Vegetable Powders and Extruded Snacks Made from Carrots and Broccoli

**DOI:** 10.3390/foods10102298

**Published:** 2021-09-28

**Authors:** Danyang Ying, Luz Sanguansri, Lijiang Cheng, Mary Ann Augustin

**Affiliations:** CSIRO Agriculture & Food, 671 Sneydes Road, Werribee, VIC 3030, Australia; Luz.sanguansri@gmail.com (L.S.); li.cheng@csiro.au (L.C.); Maryann.augustin@csiro.au (M.A.A.)

**Keywords:** vegetable powder, extruded snack, broccoli, carrot, nutrition, polyphenol, β-carotene

## Abstract

Perishable fresh vegetables that do not meet cosmetic standards and by-products of processing are currently wasted. Broccoli and carrots were selected as model vegetables to demonstrate that they can be converted into nutrient-dense and shelf-stable food ingredients and formulated into convenient ready-to-eat snacks. Broccoli powder was a rich source of protein (30%) and dietary fibre (28%). Carrot powder had lower protein (6.5%) and dietary fibre content (24%) and was higher in sugar (47%) compared to broccoli powder (21%). Compared to the whole-vegetable powders, pomace powders were richer in fibre but had lower levels of total carbohydrates. There was a reduced expansion of extruded snacks with increasing levels of the vegetable component in the formulation. Processing and storage for 12 months at 25 °C or 40 °C resulted in changes in the measured soluble phenolic content. Changes during storage were dependent on the temperature and time. The changes may be in part due to the changes in the material properties of the matrix as a consequence of processing and storage, which affect extractability. The conversion of perishable vegetables and pomace into shelf-stable nutrient-dense food ingredients and products will reduce food loss and waste in the vegetable industry.

## 1. Introduction

Improved global food security requires transformation of the food system, regional economies and natural resource management [1]. In the report The Future of Food and Agriculture: Trends and Challenges [1], a reduction in food loss and waste was identified as one of the 15 trends in food and agriculture, and a major challenge was to make food systems more efficient, inclusive and resilient. Food loss and waste is a significant global issue and can occur along all parts of the food value chain. This is also a waste of the resources that have been used in food production [2]. On the First International Day of Awareness of Food Loss and Waste, the message was to reduce food loss and waste for improved food security and environmental sustainability [3]. Recovering edible food loss and conversion into food ingredients and products will be important for a more sustainable food supply and to reduce the negative effects on the environment, society and the economy [4].

Fruit and vegetable loss and waste is a significant issue. Fruits and vegetables are a rich source of macronutrients (protein, fibre, carbohydrates, fats), micronutrients (minerals, vitamins) and phytochemicals (polyphenols, flavonoids, carotenoids etc.) [5]. Fresh produce that does not meet cosmetic standards and by-products of processing may be recovered and manufactured into a range of value-added nutritional and functional food products. This allows the capture of the high content of desirable nutrients and phytonutrients in fruits and vegetables, allowing their useful return to the human food supply [6,7]. Due to the highly perishable nature of fruits and vegetables, the conversion of produce that would otherwise be lost or wasted into shelf-stable ingredients and products is necessary. Their conversion into powders is a simple way to help preserve the nutritional components in fruits and vegetables [8]. In addition, the macronutrients have a physical functional role in contributing to the textural, colour and sensory properties of a formulated food. Whole fruit and vegetable powders as well as pomace powders may be used as an ingredient in the formulation and manufacture of healthy food products [9,10,11]. For example, fruit and/or vegetable powders may be used in a range of food applications including beverages [12], soups [13], noodles [14], bakery products [15] and extruded snacks [16].

An adequate intake of fruits and vegetables is a necessary part of a healthy diet and reduces the burden of disease [17]. Broccoli and carrots were chosen as these vegetables were of interest to the Australian horticulture industry. Broccoli florets that do not meet standards of quality (e.g., under- or over-sized or with blemishes) for fresh produce for the supermarket are wasted or left in the field. Carrots that are not acceptable for the fresh produce market are often used as animal feed. There is a growing interest in the conversion of this produce into value-added products. Broccoli and carrots are a good source of basic nutrition, and they also contain bioactive components. Vegetables such as broccoli are rich in protein and fibre and many phytonutrients that are health-promoting components, including polyphenols, glucosinolates and sulforaphane [18]. Carrots are rich in fibre and contain phytonutrients such as carotenoids and phenolic compounds [19]. There are opportunities for broccoli and carrots that would otherwise be lost or wasted to be converted into shelf-stable healthy food ingredients and products. In addition, the pomace that remains after juicing may also be used for the preparation of vegetable-based products.

The conversion of perishable vegetables into powders and extruded snacks is an attractive option for adding value to vegetables that do not make it into the fresh supply chain. In this work, powders were manufactured from whole carrots and broccoli and their pomace after juicing. The effects of processing and drying on changes in the composition of selected nutrients were examined. Extruded products containing varying amounts of broccoli or carrot solids were produced from whole broccoli and carrot powders or pomace. The characteristics of the powders and the extruded snacks were examined.

## 2. Materials and Methods

### 2.1. Processing of Vegetable Powders

**Figure 1:**
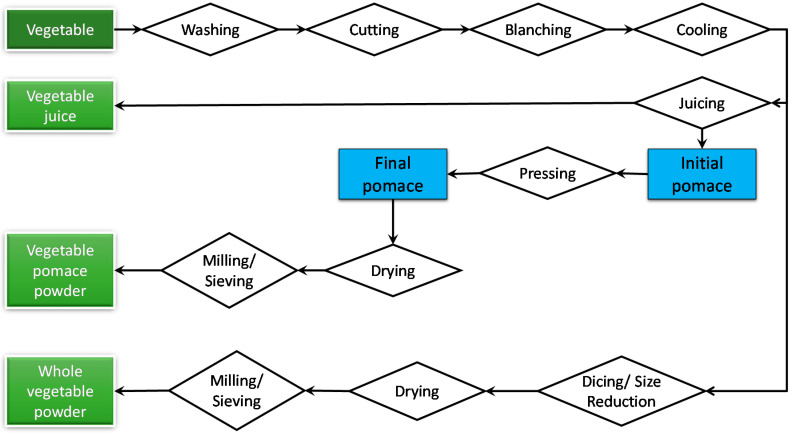
Generalised process for the preparation of vegetable powders. Trial 1: The drying method used was freeze-drying and whole-vegetable powder and pomace powder were produced. Trial 2: Fresh cut vegetables (sliced carrots and broccoli florets) were purchased from a commercial supplier and directly freeze-dried. Trial 3: Whole-vegetable powders were produced using drum drying.

Carrots (*Daucus carota*) and broccoli (*Brassica oleracea*) were obtained on three separate occasions. In Trial 1, broccoli was purchased from a local farmer/packer and carrots were purchased from a local supermarket. The stem and root ends of the carrots were trimmed, while for broccoli, the leaves were removed from the broccoli heads. Whole trimmed carrots and broccoli heads were cut into quarters and steam-blanched in a combi-oven (Rational Combi-Dämpfer CCC, Landsberg am Lech, Germany) with the oven set at 100 °C. The vegetables were placed on perforated trays in a single layer and held in the combi-oven for ~2 min at 100 °C, removed from the oven and submerged in ice water, and left until cooled to room temperature (~22 °C). The blanched vegetables were split into two parts. One part was diced into 1 cm size, using a GEODICER MP-109 Vegetable Slicer, and freeze-dried to obtain whole-vegetable powders. The other part was put through a bench top juicer (Nutrifaster Ruby 2000/MKII Juice Extractor, Australia) to remove the majority of the juice. The initial pomace obtained was transferred into a manual juice press to remove the remaining juice and a much drier vegetable pomace was obtained. The final vegetable pomace was freeze-dried to obtain vegetable pomace powders (Figure 1). In Trial 2, fresh cut vegetables (sliced carrots and broccoli florets) were purchased from a commercial supplier and directly freeze dried. In Trial 3, vegetables were purchased and processed as in Trial 1 and dried using a modified Buflovak lab-scale double-drum dryer (Buflovak ACL-4, Blaw Knox Co., Buffalo, NY, USA). All freeze-drying operations were carried out at a commercial facility (Bio-Tech Freeze Drying, 26 Parkhurst Dr, Knoxfield VIC 3180, Australia), and the dried vegetables were milled into powder using a hammer mill (Flamingo Hammer Mill, N173-023, Ernest Fleming Pty Ltd., Lane Cove, NSW, Australia) with an 800-micron sieve. The powders were packed (500 and 1000 g packs) into triple-laminated aluminium foil bags and stored at 4 °C until ready for analysis and for further processing.

### 2.2. Extrusion

Freeze-dried whole-vegetable powders prepared from Trial 2 and wet final pomace (i.e., after pressing of the first pomace, Figure 1) prepared from Trial 1 were used in extrusion trials. The dry-feed formulation was prepared by manually dry blending the freeze-dried whole-vegetable powder or wet pomace and rice flour containing 1% CaCO_3_ and 0.5% NaCl. The function of the CaCO_3_ was as a texture modifier while NaCl was included to enhance the flavour. The vegetable powder to rice flour ratios were 100:0, 80:20, 60:40, 40:60, 20:80 and 0:100. When wet pomace was used, the maximum pomace that could be incorporated into an expanded snack was about 3% (on a dry weight basis). Higher levels of addition of wet pomace in the formulation are not possible as the feed becomes too runny for extrusion, and for higher vegetable content in snacks, it is necessary to use pomace powders. The formulations used for the production of extruded snacks are given in Table 1. The extruded snacks were produced on a laboratory-scale twin-screw extruder (DSE32-II, Jinan Kredit Machinery Co., Ltd., Shangdong, China). The temperature profile along the barrel from feed to die was set to 30, 60, 100 and 140 ℃ respectively. The feed rate and screw speed were fixed at ~10 kg/h and 230 rpm, respectively. The feed moisture content was adjusted to ~20% (wet basis) by injecting water during the extrusion with vegetable powder formulations. No extra water addition was needed when the wet pomace was used. The basic screw configuration from feed to die was built with CE/37.5/37.5/8 and CE/25/25/8 to represent 8 conveying elements with a 37.5 mm length and a 37.5° helix angle and 8 conveying elements with a 25 mm length and a 25° helix angle. The extruded snacks were dried in a Quantra drying oven (Qualtex Australia P/L) at 60 °C for 4 h until water activity < 0.2 a_w_.

The expansion index of the extruded products (ratio of the product diameter over the die diameter) was measured. Ten diameter measurements were randomly taken from different sections of the extruded samples with a vernier calliper for each product sample. The average diameter was divided by the die diameter (2 mm) to obtain the expansion index.

### 2.3. Chemical Analyses of Raw Materials and Processed Vegetable Products

#### 2.3.1. Compositional Analysis

The nutritional composition of all samples was analysed at an ISO-accredited national testing laboratory (National Measurement Institute, Port Melbourne) using standard techniques for the analysis of food materials and products, which were moisture [20], protein [21], fat [22], ash [23] and sugar [24]. Carbohydrates were calculated by subtracting from 100 the quantity expressed as a percentage of moisture, protein, fat, ash and total dietary fibre. The energy was calculated by multiplying energy factors from Standard 1.2.8 of the Australia New Zealand Food Standards Code [25] by the determined quantity of a food component. The results are expressed as the measured value with the precision of the measurement.

#### 2.3.2. Total Soluble Phenolics

The total soluble phenolics (TSP) was determined using the Folin–Ciocalteau method [26]. Briefly, 6 g of the wet pureed sample or 1.5 g of the dry powder sample was weighed in a 100 mL glass bottle, and extraction solvents were added. Fifty millilitres of 80% methanol with 1% formic acid in Milli-Q grade water extraction solution was then added. The mixture was either stirred for 1 h (Trial 1) or was placed in a cool room overnight (~4 °C) with constant magnetic stirring (for Trials 2 and 3 and samples from storage trials). There was no difference in results when different extraction times were used. The mixture was then sonicated at 20 kHz (FXP range, Unisonics Australia) for 5 min then mixed using Ultra-turrax at 16,000 rpm for 1 min and left to settle. The supernatant was vacuum filtered through a Whatman medium-speed filter paper (e.g., No 2). The residue in the bottle was then mixed with 40 mL of the extraction solution and repeated as above. The bottle and filter were rinsed using 10 mL of the extraction solution. The combined filtrate was transferred to a 100 mL glass flask and topped up to the mark with the extraction solution. The extract was filtered through a 0.22 μm filter and stored in 2 mL vials until ready for UV-Vis spectrometer measurement.

A 50 µL sample extract in a 2 mL UPLC vial was transferred to a 15 mL centrifuge tube using a pipette and then 250 µL of 2N the Folin–Ciocalteu reagent and 3.0 mL MQ water were added; it was vortexed for 10 s, and then 1 mL of a 15% Na_2_CO_3_ solution was added. Finally, the solution was brought up to 5 mL by adding 750 µL of distilled water, and was vortexed for another 10 s. The mixture was incubated at room temperature for at least 1 h and centrifuged at 10,000× *g* force. Then 3 mL of the supernatant was transferred to a cuvette and the absorbance (ABS) was read at 765 nm using a UV-Vis spectrophotometer (UV-1700, Shimadzu). The total soluble phenolics was assessed by the Gallic acid standard ABS–concentration curve. The total soluble phenolics (TSP) was expressed as Gallic acid equivalents (GAE) in µg/g.

Dried powders and extruded products were packed in triple-laminated aluminium foil bags and stored at 25 °C and 40 °C for 12 months. Samples were taken at T = 0 and at 3-month intervals and stored at −18 °C until ready for analysis.

#### 2.3.3. Total Carotenoids Content

The total carotenoid content was determined using the procedure of Biswas et al. [27]. The vegetables were either pureed or dried into a powder and passed through a 500 µm sieve before analysis. Briefly, 0.5 g of puree or 0.1 g of powder were weighed in a 20 mL glass tube. Then 5 mL of chilled acetone was added and shaken for 15 min at 4 °C. Then the mixture was vortexed at high speed for 3 min. The mixture was centrifuged at 1370× *g* force for 10 min. The supernatant was transferred into a 10 mL glass flask. The residue was re-extracted with 5 mL of acetone followed by centrifugation once again. Both supernatants were pooled and topped up to the mark with acetone, then passed through a 0.45 μm syringe filter into a glass cuvette. The absorbance of the extract was determined at a 449 nm wavelength in a UV-Vis spectrophotometer. All the extractions were carried out in duplicate. The total carotenoid concentration was determined from a standard curve using β-carotene.

## 3. Results and Discussion

### 3.1. Composition of Vegetable and Pomace Powders after Manufacture

The composition of the whole-vegetable powders and vegetable pomace powders is presented in Table 2. Whole broccoli powder was a rich source of protein (30%) and dietary fibre (28%). Whole carrot powder had lower protein (6.5%) and dietary fibre content (24%) and was higher in sugar (47%) compared to broccoli powder (21%), on average. Compared to the whole-vegetable powders, pomace powders are richer in fibre but have lower levels of total carbohydrates, due to the removal of soluble solids with the juice. Other work in our laboratory on the compositions of juice and pomace fractions obtained from broccoli (leaves and stems) showed that the composition of the unfractionated broccoli (leaves and stems) was different to those of the juice and pomace fractions, as the components are partitioned differently into the juice and pomace fractions [28]. The juicing conditions and the extent of further pressing can also influence the composition of the pomace.

There were slight differences in the composition of the whole vegetable powders produced in different trials (Table 2). The maturity of the vegetables and the extent of trimmings in commercial operations can contribute to differences in composition. The drying method is not expected to have a significant effect on the gross composition on a dry basis.

The inherent composition of each of the vegetables obtained across the season, agronomic conditions and varietal differences govern the composition of the final powder [29], and as a result, differences in composition between various reports are to be expected. For example, the USDA nutrient database provides the composition for broccoli (89.3% moisture, 2.82% protein, 0.37% fat, 2.6% fibre, 1.7% sugar) and carrot (88.3% moisture, 0.93% protein, 0.37% fat, 2.8% fibre, 4.7% sugar) (https://fdc.nal.usda.gov/ndb/, accessed on 13 January 2021) [30].

### 3.2. Effect of Processing on Measured Contents of Total Soluble Phenolics

There is variation in the composition of the powders produced on different trials after manufacture, as discussed above. In addition, post-harvest storage conditions influence the content of the phytonutrients present [31]. Therefore, comparisons of the effects of processing can only be compared within a trial.

**Figure 2:**
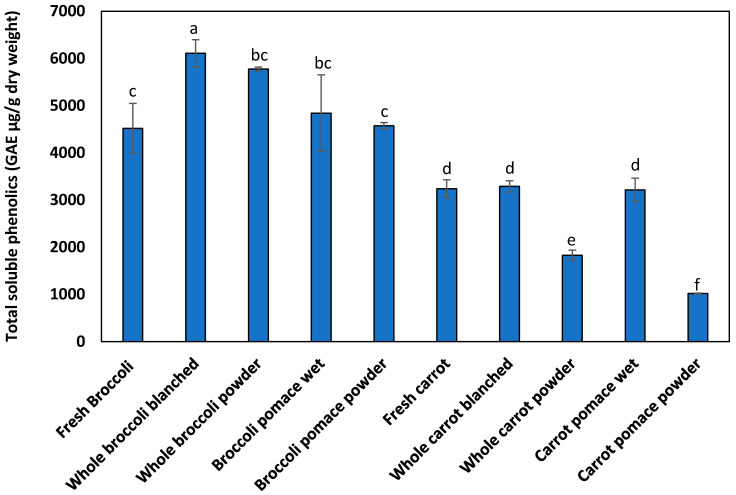
Total soluble phenolics of raw and processed vegetable biomass (Trial 1—powders freeze dried). Different lowercase letters indicate that there is a significant difference, *p* < 0.05.

Figure 2 shows the changes in total soluble phenolics during processing in Trial 1. The TSP of the fresh broccoli was 4519 µg/g GAE (dry weight) while that of the carrots was 3241 µg/g GAE (dry weight). The TSP of the whole broccoli and whole carrot after blanching was 6107 µg/g GAE (dry weight) and 3290 µg/g GAE (dry weight), respectively (Figure 2). The increased TSP on blanching is likely to be due to the breakdown of the plant cell wall structure, which made polyphenols more extractable from the blanched material [32]. Before drying, wet broccoli pomace had a lower TSP than the whole blanched wet broccoli while TSP for wet carrot pomace was similar to whole blanched wet carrot, on a dry weight basis. After drying, the TSP of the whole broccoli powder and whole carrot powder was 5775 µg/g GAE (dry weight) and 1828 µg/g GAE (dry weight), respectively, which was significantly lower than the corresponding wet materials before drying. TSP represents total soluble phenolics and it is possible that the observed decrease could be due to changes induced on the structure of the matrix during drying, which affected its extractability by the solvent. The TSP of the broccoli pomace powder and carrot pomace powder was 4572 µg/g GAE (dry weight) and 1018 µg/g GAE (dry weight), respectively. The freeze-dried powders (either whole vegetable or pomace) had a lower TSP than before drying.

In the case of broccoli, when the freeze-dried broccoli powders were produced from commercially sourced pre-cut vegetables (Trial 2), the TSP was 8548 µg/g GAE (dry weight). In Trial 3, the TSP content of the fresh broccoli before processing was 7930 µg/g GAE (dry weight) and that of the drum-dried broccoli powder was 8440 µg/g GAE (dry weight). Apart from the fact that there would be variation due to the different batches of broccoli used, another factor that influences phenol content is the processing steps applied before drying (e.g., cutting, heat treatment and pureeing). Processing steps, which result in the release of enzymes involved in phenolic synthesis and oxidation, can increase phenol content up to ~50% [33]. Depending on the blanching pre-treatment and dehydration process applied, phenolic compounds were 2203–15,987 µg/g dry weight [34]. These authors also reported that with blanching before freeze-drying, higher amounts of phenolic compounds were obtained where individual compounds were measured. In our study, blanching of vegetables also resulted in higher TSP for broccoli, but in carrots, blanching did not have an effect.

For carrots, as with broccoli, differences in TSP were observed between trials. In Trial 2, where commercially sourced fresh-cut carrots were used, freeze-dried powders had 1099 µg/g GAE (dry weight). In Trial 3, the TSP of fresh carrots before processing was 2300 µg/g GAE (dry weight) while that for drum-dried carrot powder was 1960 µg/g GAE (dry weight). Variation in TSP is expected for different batches of carrots and is also dependent on how they are processed. For example, wounding by the shredding of carrots followed by storage for 48 h results in increased levels of total soluble phenolics (from an initial value of 176 to 418 µg/g fresh weight) [35].

It is interesting to note that processing increased the TSP in broccoli. However, losses in TSP were observed in carrots due to processing. It is noted that freeze-dried carrot powder lost 43% of TSP (Trial 1), and only experienced a 15% loss in TSP for drum-dried carrot powder. This is due to the long duration of exposure of the product (48 h) to the environment while at a low temperature during freeze drying, compared to the short duration of exposure of the product (several minutes) during drum drying but at a high temperature. These results agree with the observation by Bhatta et al. [36] who reported lower retention of total polyphenol content in freeze-dried maple sugar than that of vacuum double-drum-dried sugar.

Changes in the measured TSP during processing are affected by many factors including degradation or conversion of polyphenols during processing or changed the binding of polyphenols to the matrix during drying, which affects extractability with a solvent. TSP has been used in industry as a rapid measure of total polyphenols. However, changes in individual polyphenols and other components can also affect the apparent TSP obtained. Nevertheless, they are a useful indication of changes in extractable phenols due to processing.

### 3.3. Effect of Processing on Measured β-carotene Content

**Figure 3:**
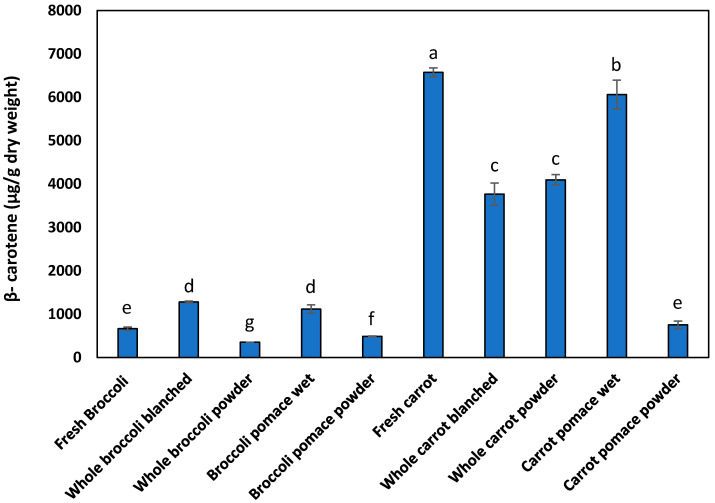
Carotenoid content of raw and processed vegetables (Trial 1—powders freeze dried). Different lowercase letters indicate that there is a significant difference, *p* < 0.05.

As with the effects of processing on total soluble phenolics, the effects of processing on β-carotene can only be compared within a trial. Figure 3 shows the changes in the measured β-carotene content of vegetable biomass and powders produced by freeze drying (Trial 1). The β-carotene content of fresh broccoli was 664 µg/g dry weight, while the β-carotene content of fresh carrots was 6574 µg/g dry weight. Whole broccoli (fresh, blanched wet or powder) contained a lower amount of β-carotene compared to whole carrots (fresh, blanched or powder) on a dry weight basis. The variation in β-carotene content in broccoli has been reported to be 370–2420 µg β-carotene/g, fresh weight, with an average value of 890 µg β-carotene/g, fresh weight [37]. For carrots, these variations in β-carotene contents have been observed with different seasonal and agronomic conditions, with up to 40 mg/100 g fresh weight (~4000 µg/g dry weight, assuming 10% moisture) across various varieties [38].

Blanching resulted in a significant loss in the β-carotene content of carrots, as might be expected. However, an apparent increase in β-carotene content was found when blanching broccoli, a result that is possibly due to altered extractability. There was a loss of β-carotene in whole-broccoli powder after blanching and drying (46% loss). With whole carrot powder, there was also a loss of β-carotene after blanching and drying (38% loss). A significant amount of β-carotene was also found in the wet pomace of the vegetables. The dried broccoli pomace powders (488 µg/g dry weight) had higher β-carotene contents than the whole broccoli powder (355 µg/g dry weight). However, dried carrot pomace powder (755 µg/g dry weight) had a lower β-carotene content than whole carrot powder (4096 µg/g dry weight).

Carotenoids are affected by variety and agronomic conditions, post-harvest storage and food-processing operations. Many food-processing operations that involve heat, especially in the presence of light, leads to the degradation of carotenoids. A recent review discusses the variety of factors that affect carotenoids content in plants during cultivation and upon processing [39].

However, the changes in the measured β-carotene during freeze drying of blanched whole vegetables and pomaces suggest that there could be artefacts introduced in the measurement. It is possible that the observed value is due to a combination of the true loss of carotenoids due to degradation, the differences in extractability from different matrices and possibly interference due to the formation of brown Maillard products (which are co-extracted). The interference due to Maillard products are expected to be greater in whole vegetables and where the sugar content of the vegetable is higher (i.e., carrot compared to broccoli, whole vegetable compared to pomace). Differences in the observed stability of carotenoids could also be due to differences in the stability of the individual carotenoids present in broccoli and carrots.

### 3.4. Properties of Extruded Snacks Containing Broccoli or Carrot

#### 3.4.1. Physical Properties

**Figure 4:**
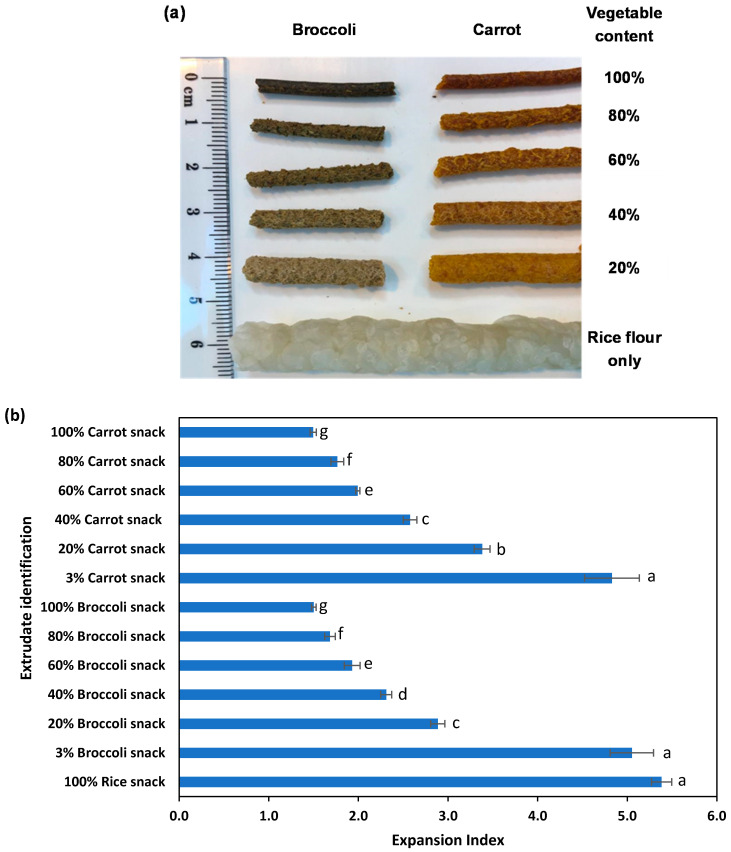
Extruded snacks containing various ratios of the whole-vegetable powder: Rice flour using powders from Trial 2 and pomace from Trial 1. (**a**) Images of snacks and (**b**) expansion index (error bar is the standard deviation of 10 measurements). Note: 3% Carrot snack and 3% Broccoli snack were made from wet pomace (from Trial 1) and rice flour. Different lowercase letters indicate that there is a significant difference, *p* < 0.05.

A photo of the extruded products containing different levels of whole broccoli or carrot powders is shown in Figure 4a. The expansion index is a measure of the density and texture of the final extruded product. A higher expansion index relates to higher sample porosity, which results in a crunchier texture. The extrudates containing broccoli or carrot powders had significantly lower product expansion, with the expansion decreasing as the amount of vegetable powder in the final product increased. The decrease in the expansion index was directly correlated with the amount of broccoli or carrot powders added, from 3% to 100% (dry basis), to the final extruded product (Figure 4b). This is due to the reduction in starch content and increased fibre content in the formulation as the vegetable powder is used to replace the rice flour in the formulation [40].

While it is possible to use pureed vegetables for extrusion, it is only possible to add ~2.5% and 3.0% vegetable biomass content (dry weight) in extruded snacks when the initial pomace (after first juicing) or final pomace (after juicing and pressing) are formulated with rice flour and extruded. The expansion index of extrudates with added wet vegetable pomace (3% dry weight) were 5.05 ± 0.24 and 4.83 ± 0.31, respectively, for carrots and broccoli (Figure 4b), which were slightly lower than the expansion index for the 100% rice snack (5.38 ± 0.11).

#### 3.4.2. Nutritional Properties

The use of vegetable powders enables the production of nutritious snacks as the vegetable components are in a concentrated form (Table 3). Table 3 shows the analysed nutritional composition of selected snacks, which correlates well with the calculated values.

#### 3.4.3. Analysis of Total Soluble Phenolics of Powders and Snacks during Storage

**Figure 5:**
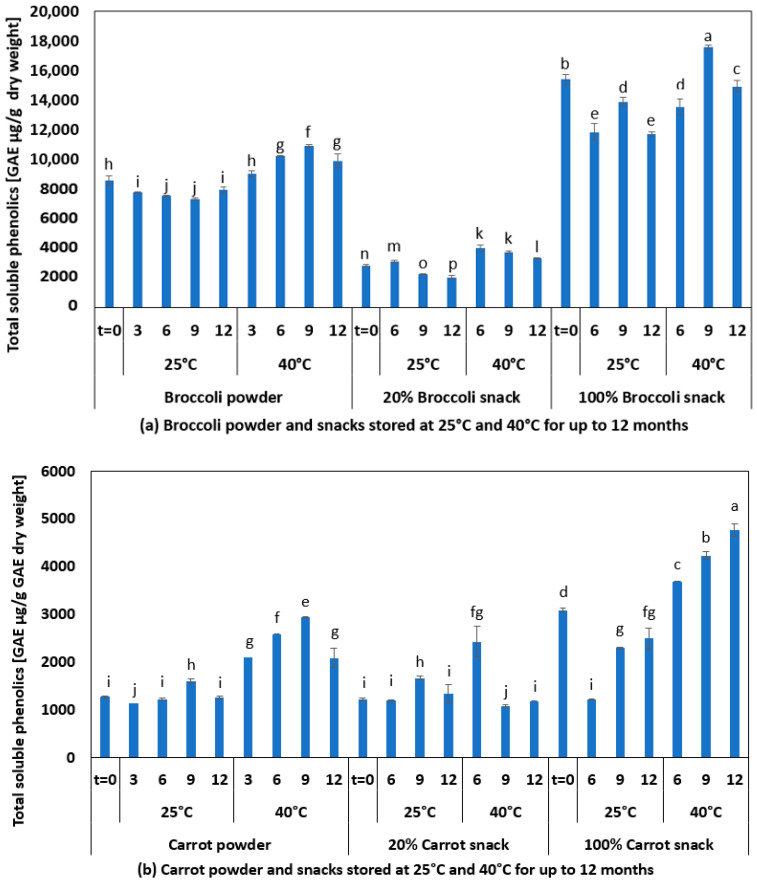
Changes in measured total soluble phenolics during the storage of powders and extrudate: (**a**) Broccoli powder and snacks, (**b**) carrot powder and snacks. Different lowercase letters indicate that there is a significant difference, *p* < 0.05.

The TSP for broccoli powder and snacks is shown in Figure 5a and for carrot powder and snacks is shown in Figure 5b. For both broccoli and carrot, TSP measured in GAE equivalents were higher in extruded samples than in the base powder (comparing the powder with 100% vegetable snacks). During the storage of broccoli products at 25 °C, there was a general reduction of TSP over 12 months of storage (72–93% remaining), whereas at 40 °C, there was a 97–118% change compared to the corresponding starting material. During the storage of carrot products at 25 °C, there was a change in TSP over 12 months of storage (81–114% remaining), whereas at 40 °C there was a 97–189% change compared to the corresponding starting material.

These results need to be interpreted with caution and should be verified using the HPLC method to quantify the individual polyphenols in order to understand which polyphenolic compounds are decreasing or increasing and what new compounds are being formed during storage that react with the Folin reagent. During storage at 40 °C, some Maillard reaction products are expected to be formed, which may have resulted in the increase.

## 4. Conclusions

Broccoli and carrot and their corresponding pomace after juice extraction and pressing were processed and dried to become shelf-stable, powder, food ingredients. Pomace powders were richer in fibre but had lower levels of total carbohydrates than the corresponding whole-vegetable powders. The use of powder in place of fresh vegetable ingredients can enhance the nutritional properties of extruded snacks as it allows the incorporation of more vegetable solids in the snack. However, an increase in the vegetable content of the formulation resulted in reduced expansion after extrusion. Providing vegetable powders and extruded snacks high in vegetable content in a convenient shelf-stable format increases the vegetable product offering for consumers. This will contribute to increased consumer choice and vegetable consumption. The production of shelf-stable vegetable powders and products will also reduce the amount of second-grade vegetables and by-products from juicing discarded as food waste.

## Figures and Tables

**Table 1:** Powdered formulation mixtures for extrusion (powder from Trial 2 and pomace from Trial 1).

Sample Code	Wet Final Pomace (%, dry basis)	Freeze-Dried Whole-Vegetable Powder (%)	Rice Flour (%)
Extruded-3	3	0	97
Extruded-20	0	20	80
Extruded-40	0	40	60
Extruded-60	0	60	40
Extruded-80	0	80	20
Extruded-100	0	100	0

**Table 2:** Typical composition of powders produced from Trials 1, 2 and 3.

Parameter	Whole Broccoli			Whole Carrot			Pomace	
	Trial 1	Trial 2	Trial 3	Trial 1	Trial 2	Trial 3	Broccoli	Carrot
Energy (kJ/100 g)	1110	1250	1150	1220	1280	1300	1120	1220
Moisture (g/100 g)	10.6	8.7	10.5	7.3	6.9	5.5	7.9	5.7
Protein (g/100 g)	30.4	31	29.3	6.7	6.3	6.5	29.2	6.0
Fat (g/100 g)	0.8	2.9	0.9	0.7	1.0	0.8	2.8	0.8
Ash (g/100 g)	9.3	7.8	8.0	7.4	6.8	7.3	7.4	5.8
Carbohydrate (g/100 g)	19.0	24	23	51.0	56	58	15.1	48.9
- Sugars (g/100 g)	18. 0	24	21	41.0	52	49	14.0	37
Sodium (mg/100 g)	370	39	230	980	1100	1100	280	640
Dietary Fibre (g/100 g)	29.7	26.1	28.2	27.1	23.1	21.8	37.6	32.4

Note: The measurement precision for the various analysis are as follows: Energy ±40 kJ/100 g, moisture ±0.2 g/100 g, protein ±4 g/100 g, fat ±0.2/100 g, ash ±0.1/100 g, carbohydrate ±2 g/100 g, sugar ±0.2 g/100 g.

**Table 3:** Nutritional composition of extruded products containing whole broccoli and carrot powder ingredients.

% Vegetable in Formulation	100% Broccoli Snack	20% Broccoli Snack	100% Carrot Snack	20% Carrot Snack
Energy (kJ/100 g)	1230	1520	1260	1530
Protein (g/100 g)	29.9	13.2	4.3	7.6
Fat (g/100 g)	3.5	1.5	1.3	0.9
Ash (g/100 g)	7.4	3.3	6.5	3.1
Carbohydrate (g/100 g)	23.0	70.0	56.0	78.0
- Sugars (g/100 g)	12.0	4.1	47.0	11.0
Sodium (mg/100 g)	58	200	1000	420
Dietary Fibre (g/100 g)	24.8	6.8	23.7	4.7

Note: The measurement precision for the various analysis are as follows: Energy ±40 kJ/100 g, moisture ±0.2 g/100 g, protein ±4 g/100 g, fat ±0.2/100 g, ash ±0.1/100 g, carbohydrate ±2 g/100 g, sugar ±0.2 g/100 g.
